# Marital experiences and depressive symptoms among older adults in rural South Africa

**DOI:** 10.1016/j.ssmmh.2022.100083

**Published:** 2022-03-02

**Authors:** Elyse A. Jennings, Chido Chinogurei, Leslie Adams

**Affiliations:** a Harvard Center for Population and Development Studies, Harvard T.H. Chan School of Public Health, USA; b School of Public Health and Family Medicine, University of Cape Town, South Africa; c Department of Mental Health, Johns Hopkins Bloomberg School of Public Health, Baltimore, MD, USA

**Keywords:** Marriage, Marital transitions, Depression, Mental health, South Africa, Apartheid

## Abstract

This paper advances the understanding of how marital transitions may influence mental health by investigating these associations among a population of rural, Black South Africans aged 40+ that was directly impacted by apartheid. Using two waves of data from 4,176 men and women in Health and Aging in Africa: A Longitudinal Study of an INDEPTH Community in South Africa (HAALSI), we investigated associations between marital experiences and depressive symptoms, by gender, and explored whether economic resources is a moderator of these associations. We found that experiencing a marital dissolution was associated with more depressive symptoms than remaining married for both men and women. We also found that men, but not women, report greater depressive symptoms if they remained separated/divorced, remained widowed, or remained never married between waves. We found no evidence that a decline in wealth moderated the impact of marital dissolution on depressive symptoms for women or men. These findings suggest that the documented benefits of marriage for mental health, and differences by gender in those benefits, may extend to older, rural South Africans, despite the unique experiences of this population.

## 1. Introduction

Marriage has been found to offer mental health advantages, relative to singlehood, widowhood, or divorce (Barrett, 2000; Carr & Springer, 2010; Sasson & Umberson, 2013; Waite & Gallagher, 2002). Evidence suggests that men and women differentially benefit from marriage and marital transitions (Sasson & Umberson, 2013; Waite, 1995), and these gender differences may translate into different impacts on mental health for women and men (Scott et al., 2010; Simon, 2002). However, our understanding of this relationship among older adults, and especially those in low and middle-income countries (LMICs) and non-White majority populations, is limited. Marriage can have unique meaning and value across settings (Cherlin, 2012; Thornton, 2001), and setting-specific gender dynamics may lead to unique influences of marriage and marital transitions on men’s and women’s mental health.

There is evidence that mental health may decline with age among sub-Saharan African (SSA) populations (Kohler et al., 2017). Older South African adults face significant challenges in combating both communicable and non-communicable disease, as a high proportion of the population have been infected with HIV or suffer from other morbidities (e.g., hypertension, diabetes, and heart disease) (Gaziano et al., 2017; Nojilana et al., 2016; Wade et al., 2021). The number and proportion of older adults in SSA is projected to increase substantially in the coming years and decades, exacerbating the need to understand health issues facing his vulnerable group (GBD 2015 DALYs and HALE Collaborators, 2016; Kohler et al., 2017). Among Black South Africans, older age is even more consequential: Older adults lived a greater portion of their lives under the systematic racism imposed by apartheid, before it began to be dismantled in the early 1990s. The effects of apartheid have been long lasting, impacting marital patterns decades after it formally ended (Amoateng and Heaton (eds.) 2007; Budlender & Lund, 2011). These dynamics, combined, make understanding the impacts of marital experiences on mental health among older South Africans a public health priority (Meyer et al., 2019).

We use two waves of data from Health and Aging in Africa: A Longitudinal Study of an INDEPTH Community in South Africa (HAALSI) to investigate associations between marital experiences (i.e., marital stability or transitions) and depressive symptoms, within a four-year time span. Our analyses are stratified by gender and examine whether economic resources moderate these associations for men and women. The HAALSI data represent a cohort of Black Africans living in rural South Africa, who were aged 40 or older at baseline (in 2014/2015). By expanding the understanding of associations between marriage and mental health to this population, our study advances knowledge of how these associations operate across different contexts, with particular consideration for the post-apartheid context.

### 1.1 Setting

The study area for this investigation is a cluster of villages in and near Agincourt: A rural, low-income sub-district in Mpumalanga Province, located in the northeastern part of South Africa. Agincourt is in an area of forced racial segregation during apartheid, to which Black South Africans belonging to the Shangaan ethnic group were forcibly moved (Blalock, 2014). Mpumalanga is the second most homogenous province in South Africa, with over 90% of residents identifying as Black African (Statistics South Africa, 2018).

The oppressive nature of political repression and economic exploitation experienced by this population resulted in pervasive negative consequences for mental health. These consequences include not only differential care for psychiatric disorders and psychological distress, but a paucity of research focused on the extent to which racialized policies influenced the mental health of Black South Africans (Adams et al., 2020; Dommisse, 1986; Swartz, 1991; Turton & Chalmers, 1990). In recent years, researchers have examined the influence of perceived chronic and acute exposure to racial discrimination in South Africa, with studies finding strong associations between both forms of racial discrimination and elevated risk of any 12-month DSM-IV disorder among historically marginalized groups in South Africa compared to Whites (Moomal et al., 2009; Williams et al., 2008). Within Mpumalanga province, the lifetime prevalence of DSM-IV (Samuel B. Guze, 1995) mood disorders was 9%, compared with the national average of 9.7% (Herman et al., 2009). In terms of severity of DSM-IV disorders in a 12-month period, this region also reports significantly lower provincial rates of mental health disorders classified as moderate and severe, but higher rates of disorders classified as mild, compared to the remaining country provinces. The prevalence of mental health in other aging SSA populations can provide insight into this Agincourt population: In rural Malawi, relatively few adults aged 45 and older (about 2%) were found to have severe or moderate depression and/or anxiety (Kohler et al., 2017). These mental health symptoms were found to increase with age—unlike patterns typically found in high income settings—and were more prevalent among women than men (Kohler et al., 2017). The high prevalence of HIV in SSA, including South Africa (Wade et al., 2021), may take a unique toll on mental health (Hsieh, 2013).

Individuals’ marital histories are often complex in SSA settings (Boileau et al., 2009; Budlender et al., 2004; Myroniuk et al., 2021; Reniers, 2003). In South Africa, these complexities and the current marital and gender dynamics are uniquely tied to the legacy of apartheid. During apartheid, Black African adults were not permitted to stay in urban areas without employment and accommodation, and men were often forced to leave their families to live in single-sex hostels while they worked in the city (Hosegood et al., 2009). This resulted in the splitting of families and the entering of women into the labor force in greater numbers, which, in turn, led to a decline in marriage rates, a rise in separation and divorce, and an increase in female-headed households among rural Black populations (Budlender & Lund, 2011; Hosegood et al., 2009).

Marriage rates remain lower in South Africa than in other SSA countries (Clark & Brauner-Otto, 2015). For example, based on estimates from the 2016 Demographic and Health Survey, 61% of women and 71% of men in South Africa aged 25–29 were never married (National Department of Healthand, 2019). By comparison, among those aged 25–29 in Zimbabwe in 2015, 9.5% of women and 31% of men were never married; in Nigeria in 2018, those percentages were 13% among women and 49% among men (National Population Commission - NPCand, 2019; Zimbabwe National Statistics Agency and ICF International 2016). Similarly, in the U.S., 23% of men and 17% of women over age 25 were never married as of 2012 (Wang & Parker, 2014). South African marriage rates continued to decline even after the fall of apartheid (Hosegood et al., 2009). The decline has been attributed to increased education and more economic opportunities for women (Garenne et al., 2001; Kalule-Sabiti et al., 2007), and to economic constraints for men (Posel et al., 2011).

Marital trajectories among this population often involve multiple unions and dissolutions (Amoateng et al. 2004; Hosegood et al., 2009). As of 2014/2015, about 13% of Agincourt residents aged 40 or older were currently separated or divorced. Due to the high mortality among this population, rates of widowhood are relatively high, especially among women. About 30% of Agincourt residents aged 40 or older (and 47% of women, in particular) were currently widowed in 2014/2015. Twenty-three percent of this sample had been married more than once.

## 2. Conceptual framework

### 2.1 Marriage and mental health

Past research suggests that people who are married face greater health advantages compared with their single counterparts (Carr & Springer, 2010; Waite & Gallagher, 2002), and many studies offer evidence that the benefits of marriage extend to mental health (Braithwaite & Holt-Lunstad, 2017; Simon, 2002; Strohschein et al., 2005; Uecker, 2012). Although this could be due to selection of mentally healthier people into marriage (Braithwaite & Holt-Lunstad, 2017; Uecker, 2012)—just as physically healthier people are selected into marriage (Lipowicz, 2014; Waldron et al., 1996)—a few studies have found that the effects exist even after accounting for this selection (Frech & Williams, 2007; Lamb et al., 2003; Marcussen, 2005; Simon, 2002).

In Western settings, just as stable marriage can have positive impacts on mental health (Barrett, 2000; Uecker, 2012), marital dissolution can have relatively negative impacts (Lee & DeMaris, 2007; Marks & Lambert, 1998; Sasson & Umberson, 2013; Waite et al., 2009). Both divorce and widowhood may affect depressive symptoms via the stressors that they involve. In the case of divorce, stressors might come from adjustment to life after marriage, including adjustment to new economic circumstances (Bröckel & Andreß, 2015; Waldron et al., 1996). Similarly, the stressors involved in caregiving that often precedes widowhood can have a detrimental impact on mental health (Carr & Springer, 2010). At the same time, the loss of that caregiving role combined with the loss of a spousal companion can cause particular emotional stress (Lee & DeMaris, 2007; Sasson & Umberson, 2013; Schaan, 2013). These negative impacts associated with widowhood may be stronger in the short term and dissipate over time (De Leon et al., 2009; Harlow et al., 1991). Moreover, remarriage can offer benefits to mental health over remaining divorced or widowed, but may still be a disadvantaged status relative to remaining in a stable marriage (Barrett, 2000; Carr & Springer, 2010; Kim & McKenry, 2002).

There are apparent racial differences in the impacts of marital status on mental health. A U.S.-based study found that early entry into marriage was associated with greater depression for African American men, but not for White men or for women (Mullan Harris et al., 2010). On the other hand, earlier studies pointed toward the notion that marital status may have a greater impact on the mental health of White people than their Black counterparts (Williams et al., 1992), and that Black families are not as negatively impacted by singlehood or marital dissolution (McKelvey & McKenry, 2000; Pudrovska et al., 2006). Reasons for these racialized differences are not well understood, but may be linked to the strong social and family ties in African American communities that extend beyond the nuclear household (Cross et al., 2018; Gerstel, 2011). These extended family networks, which are also pervasive in the Agincourt setting (Schatz et al., 2015), may offer important support to buffer against negative impacts of marital transitions.

Although more limited, studies based in SSA—namely, Malawi—have provided mixed results in the relationship between marital status and health outcomes among older adults. In some cases, marital dissolution has been found to be detrimental to self-rated health and psychological well-being (Clark et al., 2020; Myroniuk, 2016). However, the magnitude of associations between marital dissolution and self-rated health was weaker than anticipated, suggesting that people in such settings who survive to older ages may be selective on factors that make them more resilient against the potential negative impacts of marital dissolution (Myroniuk, 2016). These studies offer important insight into the interrelationships between marriage and health in SSA settings, but they are limited in their sample sizes and their ability to distinguish between effects of widowhood versus divorce (Clark et al., 2020; Myroniuk et al., 2021).

### 2.2. The role of gender in marriage and mental health

Studies among Western populations offer mixed evidence of gender differences in the health consequences of marriage and marital transitions. In some work, men have been found to experience greater health benefits from marriage than women, possibly because they receive more emotional support and encouragement of healthy behaviors—typically taken on more by wives than husbands (Carr & Springer, 2010; Umberson, 1992). Likewise, studies have shown that unmarried women fare better on psychological well-being than unmarried men (Hsu & Barrett, 2020; Marks, 1996), and that remarriage may be more advantageous for men’s psychological well-being than women’s (Williams, 2003). However, other studies challenge the idea that women in non-marital statuses have better psychological well-being than men: Kim and McKenry (2002) found that never married women fare worse than their male counterparts, and Marks and Lambert (1998) found that women fare better when staying married than becoming divorced or widowed, compared with men. If women fare worse than men when unmarried, this may be tied to the economic advantage that marriage disproportionately affords women (Sasson & Umberson, 2013). In the case of divorce, it may be tied to the greater burden of adjustment to economic status after divorce among women than men (Bröckel & Andreß, 2015; Smock et al., 1999).

Alternatively, there is evidence that men and women are impacted similarly by marital loss, but have different psychological reactions (Simon, 2002; Sonnenberg et al., 2000), leading to different outcomes in their mental health (Carr et al., 2001; Lee et al., 2001). Still another body of research from the U.S. suggests that men and women are not differentially affected, psychologically, by marriage and marital transitions (Carr & Springer, 2010; Frech & Williams, 2007; Schaan, 2013; Williams, 2003). In combination, these studies present a mixed picture of the psychological reactions to different marital experiences of men versus women in Western, high-income settings.

The gendered impact of marital transitions is likely to be heavily influenced by setting-specific cultural and social norms. For example, studies in South Asia, where arranged marriage is still common and the nature of marriage remains highly patriarchal and patrilineal, have found that marriage may be harmful to women’s mental health (Axinn et al., 2020). Similarly, a study in Malawi found that experiencing more marital dissolutions can be beneficial to women’s mental health (Myroniuk et al., 2021). The authors offer the possible explanations that divorce may empower women in ways that benefit their mental health, or that mentally healthy women experience more marriages because they are more likely to remarry. Meanwhile, results for men more closely mirror findings in Western settings: Spending more time outside of marriage is associated with worse mental health (Myroniuk et al., 2021). On the other hand, another study in the same Malawian setting found that being formally married was associated with worse psychological well-being for men and women alike, and this was more pronounced among those in reproductive ages (Clark et al., 2020). These findings, which sometimes contradict findings in high-income settings, illustrate the importance of investigating these associations across disparate settings.

### 2.3. Marriage and mental health in rural South Africa

We extend the evidence base by testing specific hypotheses regarding the associations between marital experiences and depressive symptoms among an aging cohort of Black South Africans in Agincourt. Apartheid and its legacy are inextricably linked to the experiences of this population, and to the social construction of marriage (Amoateng and Heaton (eds.) 2007; Budlender & Lund, 2011; Hosegood et al., 2009). As such, we conceptualize the experience of apartheid and its legacy as the backdrop against which associations between marital transitions and mental health occur, rather than a variable on the pathway between these associations.

Mental health may have become less connected to marriage during apartheid and its aftermath, as spousal separation likely led to the reliance on non-spousal sources of support (Jennings et al., 2020). Moreover, with the prevalence of marital dissolution (through both divorce and widowhood), stable marriage may offer limited benefits for mental health. For instance, better mental health among married people than cohabiters in the U.S. has been attributed to the greater stability and commitment of marriage (Brown, 2000; Marcussen, 2005). Likewise, the low marital stability and permanence in Agincourt may have rendered the benefits of marriage for mental health less salient. In a similar vein, Clark et al. (2020) find that marital dissolution is less detrimental to psychological health of older than younger adults, and posit that this could be due to greater normalcy and social acceptability of being unmarried at older ages.

The gendered roles within marriage in SSA are distinct. In South Africa, many households are female-headed (Schatz et al., 2011), and the greater self-sufficiency of women may mean that the presence of a spouse is not a salient factor in women’s mental health (Hosegood et al., 2009). For men, marriage may even be related to greater depressive symptoms in this setting. In South Africa, like other parts of SSA, masculinity is tied to marriage and the family, as the patriline insures that men’s identity is passed to future generations (King & Stone, 2010). In order to marry, a man and his family must first pay lobola (the local name for bridewealth), as a part of the patrilineal custom in places like Agincourt (King & Stone, 2010). As such, the act of marrying is tied to economic status, as are the responsibilities associated with being married. A married man’s masculinity can feel especially threatened if they lack employment or wealth (Barker & Ricardo, 2005; Fry et al., 2019), as is the case for many men in Agincourt (Collinson et al., 2016). Hence, marriage may be associated with poorer mental health among men in this setting.

**H1.** Non-married (i.e., never married, separated/divorced, or widowed) and newly married (i.e., transitioned from never married to married, or from separated, divorced, or widowed to married) women have similar depressive symptoms as their counterparts in stable marriages.

**H2.** Non-married men and men who experience marital dissolution have fewer depressive symptoms than their counterparts in stable marriages, while newly married men have similar depressive symptoms as their counterparts in stable marriages.

### 2.4. Protective role of economic resources

Economic resources can play a crucial role in the relationship between marital experiences and depression (Myroniuk et al., 2021). The economic advantages to marriage are important drivers of its health benefits (Carlson & Kail, 2018; Marcussen, 2005), and may be especially salient for women in Western settings (Smock et al., 1999; Strohschein et al., 2005). In Agincourt, however, we may find limited evidence that economic resources moderate the impact of women’s marital experiences on their depressive symptoms, as these women are often financially independent from men and able to rely on extended family for financial support (Hosegood et al., 2009; Lloyd-Sherlock, 2000). Men’s depressive symptoms, on the other hand, may react to changes in economic resources that accompany marital transitions, as their economic earnings and status are often central to their identity (Barker & Ricardo, 2005; Bigombe & Khadiagala, 2003; Fry et al., 2019).

**H3.** Economic resources moderate the association between marital experiences and depressive symptoms for men but not for women in this setting.

## 3. Methods

### 3.1. Study design

We use data from Health and Aging in Africa: A Longitudinal Study of an INDEPTH Community in South Africa (HAALSI). Participants were sampled from the existing framework of the Agincourt Health and Socio-Demographic Surveillance System (Agincourt HDSS) site in Mpumalanga province. The Agincourt HDSS is part of the global INDEPTH Network, which monitors the health and population of many low and middle-income countries. Individuals 40 years and older as of July 1, 2014 and permanently living in the study site during the 12 months prior to the 2013 Agincourt census update were eligible. From these data, a sampling frame of 12,875 was identified and, using gender-specific sampling fractions to ensure a gender-balanced cohort, 6,281 were randomly selected to participate. Wave 1 interviews were conducted between November 2014 and November 2015 with 5,059 respondents, using Computer Assisted Personal Interviews (CAPI) in the local language, Shangaan. Among these respondents, 18% were in their 40s, 28% in their 50s, 26% in their 60s, 17% in their 70s, and 11% were aged 80 or older. See Gómez-Olivé et al. (2018) for more detail on the study sample and design.

Wave 2 interviews were conducted between October 2018 and November 2019, with 83% (n = 4,176) of the original respondents. Of the remaining 17%, about 12% had died, 5% refused to participate in the interview, and less than 1% were lost to follow-up. We use multiple imputation on missing values across measures (noted where relevant below) in order to retain the full wave 2 sample and to avoid bias in our analyses (Harel et al., 2017). Missing data were treated as missing at random, and imputed using the PROC MI and PROC MIANALYZE functions in SAS (Yuan, 2011). The imputation model used information from each variable in our models as well as two auxiliary variables that were found to correlate with measures requiring imputation: a latent measure of cognitive function and time since last health consultation. Ten imputed datasets were specified. We include tables in supplemental materials showing results using listwise deletion instead of multiple imputation (Supplemental Tables 1–3), as well as results weighted to adjust for mortality and other attrition (Supplemental Tables 4–6).

### 3.2. Measures

#### Depressive symptoms.

Wave 2 of HAALSI captured the full 20-item Center for Epidemiologic Studies Depression (CES-D) scale. This series of 20 items were prefaced with the introduction “Now think about the past week and the feelings you have experienced. Please tell me how often you have felt this way during the past week.” Examples of the 20-items include “I was bothered by things that usually don’t bother me” and “I felt I was as good as other people”. Response options were: “rarely or none of the time (less than 1 day),” “some or a little of the time (1–2 days),” “occasionally or a moderate amount of time (3–4 days),” and “most or all of the time (5–7 days)”. As per convention, we coded the response options on a scale from 0 to 3 (reverse coding items as appropriate to indicate symptomology, e.g. “I felt hopeful about the future”), then summed responses across the 20 items, for a possible range of 0–60 (Hann et al., 1999). We imputed data for 288 respondents (7% of the sample) on this measure (these missing cases were mostly due to interviews being completed by proxy respondents, who were not asked to report on CES-D). We use a z-standardized version of this measure, centering the mean at zero and the standard deviation (SD) at one. Although the CES-D was designed for predominantly white populations, there is evidence that an abbreviated version, which was used in wave 1 of HAALSI, is valid among the HAALSI sample (Adams et al., 2020).

#### Marital experiences.

We use a series of measures to reflect whether the respondent reported (1) being married to or living with the same partner in both waves (“remained married”), (2) entering into a new marriage in wave 2 (including those who reported being never married, separated, divorced, or widowed at wave 1 and then married or living with partner in wave 2), (3) being separated/divorced in both waves (“remained separated/divorced”), (4) being widowed in both waves (“remained widowed”) (5) experiencing a marital dissolution (i.e., a separation, divorce, or widowhood), between waves, or (6) being never married at both waves (“remained never married”). These categories were created after testing distinct categories (e.g., separation vs. divorce vs. widowhood), then collapsing categories that are both conceptually similar and were not found to have significantly different associations with our dependent variable. We coded 194 cases as missing due to illogical reporting of being married/partnered, divorced, or widowed at wave 1 and never married at wave 2. We then imputed data on a total 211 cases (5% of the sample) for marital experiences.

Our operationalization of marriage includes both married individuals and unmarried cohabiters. Although we are limited to this operationalization because “married or living with partner” was a single response option in the HAALSI survey, this approach is appropriate. There may be a tendency to otherwise underreport romantic unions, especially in rural areas, since these unions will only be socially recognized if lobola has been paid (Hosegood et al., 2009; Parker, 2015). However, a follow-up question was added in Wave 2 that captures whether current unions were formalized, either by lobola and/or at the magistrate. Only 7% of those who reported to be “married or living with partner” in wave 2 then reported that they had not formalized the union by one of these two means. This offers confidence in conceptualizing those who select this response as a fairly uniform group of mostly formalized partnerships. As such, we refer to these unions as marriages hereto forth.

#### Decline in wealth.

Economic resources were operationalized as change in a respondents’ household wealth between waves 1 and 2. We coded this measure to reflect decline in respondents’ household wealth, based on quintiles defined by the distribution of household wealth in wave 1. Using wave 1 data from the full HAALSI sample, households were ranked according to the scores from principal components analysis of household ownership of items such as televisions, refrigerators, livestock, vehicles as well as housing characteristics, type of water and sanitation facilities (Riumallo-Herl et al., 2019). This ranking was then collapsed into quintiles. We coded a measure of household wealth at wave 2, using the same cut-offs for each of the five categories that defined the five quintiles in wave 1. Next, we subtracted the measure of household wealth at wave 2 from the measure of household wealth at wave 1, and collapsed into a three category measure indicating decline in wealth: If they moved down one or more quintiles, they received a code of 1; if they moved up one or more quintiles, they received a code of −1; and if they stayed in the same quintile, they received a code of 0.

#### Controls.

To adjust for depressive symptoms at baseline, we treat the 8-item CES-D in HAALSI wave 1 as a control variable. In wave 1, this abbreviated version of the CES-D was administered, with “yes” and “no” as the response options, in order to mirror the measure used by the Health and Retirement Study (HRS) at the time (e.g., Glymour et al., 2010). Our control measure sums the symptomatic responses, ranging from 0 to 8. We imputed data on baseline CES-D for 65 cases (1.6% of the sample).

We also control for a series of demographic covariates, measured at wave 1, that may affect the associations we investigate. In particular, we account for respondent’s gender (1 = female, 0 = male), age (continuous), whether married more than once (5 cases were imputed; <1% of the sample), number of living children (9 cases were imputed; <1% of the sample), and educational attainment (series of dummy variables to indicate [1] no formal education, [2] some or completed primary education, or [3] some or completed secondary or higher education). We also control for primary employment status. These measures come from a survey item in which respondents could check as many response options as applied to them, and we code status into four dummy measures to indicate (listed in order of how we prioritized responses): (1) employed, (2) home manager (originally meant to indicate “homemaker”, but translated to be understood as “one who manages the home”), (3) retired, or (4) not working. We imputed data on employment for 11 cases (<1% of the sample).

Next, we control for physical co-morbidities at wave 1, with a measure indicating whether the respondent suffers from diabetes (either self-reported or confirmed in blood glucose), angina (self-report), and/or hypertension (from blood pressure). If a respondent is missing data on any of three possible comorbidities but suffers from any of the remaining comorbidities, then they received a code of 1. Missing data was imputed on co-morbidities for 162 cases (4% of the sample). We also account for HIV status at wave 1, with a measure indicating whether the respondent is HIV positive (from blood samples). A total of 361 cases were imputed on HIV status (9% of the sample), due to refusals or indeterminate blood tests.

We also control for household level covariates at wave 1: household size (top coded at 8) and household wealth quintiles (as described above).

Finally, we control for whether the respondent was born inside of South Africa, coded as 1 if so and 0 otherwise. Although all HAALSI respondents are Black Africans and therefore impacted by the legacy of apartheid, 32% were born outside of the country (due to the large immigrant population from Mozambique) (Kahn et al., 2012).

### 3.3. Analysis

We use the following ordinary least squares (OLS) regression equation to investigate the associations between marital experiences from waves 1 to 2 and depressive symptoms at wave 2.


$$CESD20_{it+1}=\beta_{0}+\beta_{1}NewMar_{i}+\beta_{2}RemSepDiv_{i}+\beta_{3}RemWid_{i}+\beta_{4}MarDiss_{i}\;\;+\beta_{5}RemNevMar_{i}+\beta_{6}CESD8_{it}+\beta_{7}Cov_{it}+\varepsilon_{i}$$

In this equation, *CESD*20_*it*+1_ denotes the 20-item CES-D for individual *i* at wave 2 (*t*+*1*). *NewMar*_*i*_ denotes the experience of entering into a new marriage between waves 1 and 2, *RemSepDiv*_*i*_ denotes the experience of remaining separated or divorced between waves, *RemWid*_*i*_ denotes the experience of remaining widowed between waves, *MarDiss*_*i*_ denotes the experience of a marital dissolution between waves, and *RemNevMar*_*i*_ denotes the experience of remaining never married between waves. *CESD*8_*it*_ denotes the CESD-8 at wave 1 (*t*), and *Cov*_*it*_ denotes the vector of controls, described above, for individuals’ characteristics at wave 1.

We use the following OLS regression equation to investigate the interactive effect of decline in wealth between waves and marital experiences on depressive symptoms at wave 2. *WlthChg*_*i*_ denotes the three-level measure of decline in wealth between waves 1 and 2.


$$CESD20_{it+1}=\beta_{0}+\beta_{1}NewMar_{i}*WlthChg_{i}+\beta_{2}RemSepDiv_{i}*WlthChg_{i}\;\;+\beta_{3}RemWid_{i}*WlthChg_{i}+\beta_{4}MarDiss_{i}*WlthChg_{i}\;\;+\beta_{5}RemNevMar_{i}*WlthChg_{i}+\beta_{6}NewMar_{i}+\beta_{7}RemSepDiv_{i}+\beta_{8}RemWid_{i}\;\;+\beta_{9}MarDiss_{i}+\beta_{10}RemNevMar_{i}+\beta_{11}WlthChg_{i}+\beta_{12}CESD8_{it}+\beta_{13}Cov_{it}\;\;+\varepsilon_{i}$$

In order to test the reliability of our results, we ran regression diagnostics, including the variance inflation factor (VIF), studentized residual, and Cook’s D. These diagnostics indicated that multicollinearity, outlying values, and particular observations are not affecting our results.

## 4. Results

Table 1 displays the mean values of variables in our models for women and men separately. The final column indicates the differences and significance levels for men and women on each variable. Women report an average of 0.06 SDs above the pooled sample mean, and men report an average of 0.07 SDs below the mean for depressive symptoms at wave 2. This translates to an average of approximately 15 symptoms among women and 14 symptoms among men. Women in this sample had significantly more depressive symptoms, on average, than men on both the 20-item CES-D at wave 2 and the 8-item CES-D at wave 1. Fewer women than men remained married between waves, at 31% of women compared with 60% of men. Similarly, more men than women entered into a new marriage between waves (4% versus 2%). The same percentage (9%) of men and women remained in a separated or divorced status between waves. Substantially more women than men remained widowed between waves (42% versus 10%), and slightly more women than men experienced a marital dissolution between waves (14% versus 12%). Finally, fewer women than men remained never married between waves (2% versus 5%). Additionally, the average on the measure of decline in wealth between waves was 0.05 for women and 0.07 for men, indicating that women experienced less decline (greater increase) in wealth quintiles than men did. In addition to these significant gender differences, we also find significant differences to suggest that women in our sample are slightly younger, are less likely to have been married multiple times, have fewer children, are more likely to have at least one comorbidity, are less educated, are more likely to be not working or to be managing the home (and less likely to be employed or retired), live in larger households, and are less likely to have been born in South Africa than men.^1^

Table 2 displays results from OLS regression. Model 1 tests our first hypothesis by estimating the association between marital experiences and the number of depressive symptoms at wave 2 among women. These results suggest that experiencing a marital dissolution was associated with 0.18 SDs greater depressive symptoms among women, relative to remaining married. We find no evidence that women who entered a new marriage, experienced stable separated/divorced or widowed statuses, or remained unmarried faced significantly different depressive symptoms than those who remained married between waves. This offers support for our first hypothesis.

In Model 2 we test our second hypothesis by estimating these same associations between marital experiences and depressive symptoms among men. We find that entering a new marriage is modestly significant for men, and associated with 0.23 SDs greater depressive symptoms than remaining married between waves.^2^ Remaining separated or divorced between waves was associated with 0.31 SDs greater depressive symptoms, and remaining widowed was associated with 0.20 SDs greater depressive symptoms than remaining married among men. Either experiencing a marital dissolution between waves or remaining never married were each associated with 0.33 SDs greater depressive symptoms among men, relative to remaining married. Our second hypothesis is therefore not supported.

In Table 3, we address our third hypothesis by investigating the moderating role of decline in wealth for women and men, separately. Model 1 of Table 3 is identical to Model 1 of Table 2, except that the control for baseline wealth index is removed and we now include our measure indicating decline in wealth between waves. We find no evidence that decline in wealth is significantly associated with depressive symptoms at wave 2 among women. In Model 2, we test a series of interaction terms, between each category of marital experiences and our measure indicating decline in wealth. None of the interaction terms are significant, suggesting no evidence that decline in wealth between waves moderates the association between women’s marital experiences and depressive symptoms.

Models 3 and 4 of Table 3 replicate the first two models among the sample of men. We find similar results among men: Decline in wealth is not significantly associated with depressive symptoms, and none of the interaction terms are significant. Hence, we find no evidence that a decline in wealth moderates the impact of men’s marital experiences on their depressive symptoms. Our third hypothesis is not supported.

Many of the covariates in our model were also significantly associated with depressive symptoms. Depressive symptoms at wave 1 and age were both positively associated with depressive symptoms at wave 2 for women, but non-significant for men. This finding may be due, in part, to our approach of using a shortened CES-D measure at Wave 1 to predict a more expansive 20-item CES-D measure in Wave 2. Indeed, abbreviated CES-D measures are skewed towards items that reflect negative affect, such as crying and depressed mood, which may be more likely to be endorsed by women than men (Adams et al., 2020; Lewinsohn et al., 1997). We also found that having either primary or secondary education was negatively associated with depressive symptoms for men, relative to having no education, but non-significant for women. Being employed was negatively associated with depressive symptoms for men, relative to not working, while being retired had a similar and significant impact for women. These findings related to education and employment align with extant research in South Africa that identifies these factors as protective (Ardington & Case, 2010; Hamad et al., 2008; Tomlinson et al., 2009).

## 5. Discussion

In this paper, we have investigated how experiencing different marital transitions (or stability in different marital statuses) can be associated with depressive symptoms, among a population of older Black South Africans in a low income, rural setting. This population has experienced unique challenges that have impacted both the health profile and the stability of marriage, including the prevalence of both communicable and non-communicable disease and the salience of apartheid policies and their legacy (Budlender & Lund, 2011; Gaziano et al., 2017; Wade et al., 2021). Although we focus on marital and depressive experiences within a relatively brief span of time—about four years—we are able to assess possible consequences of recent marital transitions (i.e., separation/divorce/widowhood and new marriage in the past four years), as well as marital experiences that have been stable for longer than four years. Our results suggest that men and women, alike, who experienced a marital dissolution between the two waves of our study reported a greater number of depressive symptoms, relative to their respective counterparts who remained married between waves. We also find modest evidence that men who entered a new marriage (including remarriage and first marriage) experienced more depressive symptoms than their stably married counterparts. We find stronger evidence that men who remained separated/divorced, widowed, or never married between waves experienced more depressive symptoms than their stably married counterparts. Finally, we found no evidence that a decline in wealth status between waves moderated these associations for either women or men.

Our hypotheses were developed to test the overarching expectation that marital experiences exhibit unique associations in this setting, compared with Western settings, given the unique experiences of Black South Africans during and after apartheid. However, many of our findings mirror findings in both Western and other SSA settings. This suggests that the costs and benefits of marriage and marital dissolution may share some universal characteristics across different populations.

Marital dissolution has been found to be associated with worse mental health in several settings, including the U.S. (Afifi et al., 2006; Barrett, 2000; Marks & Lambert, 1998), Europe (Richards et al., 1997; Wade & Pevalin, 2004), South Asia (Axinn et al., 2020), and Malawi (Clark et al., 2020; Myroniuk et al., 2021). In this South African setting, marital dissolution via both marital breakdown and spousal death is prevalent. This normalcy of marital dissolution led us to expect that marriage in this setting would not confer mental health benefits. For men, we went a step further to offer the hypothesis that marital dissolution may benefit their mental health, by freeing them from the hard-to-fulfill responsibility of financially supporting their spouse and children (Barker & Ricardo, 2005; Bigombe & Khadiagala, 2003). However, our results suggest that experiencing a marital dissolution, relative to remaining in a stable marriage, is detrimental to depressive symptoms for both women and men in the HAALSI sample. Marital dissolution may have distinct, but still detrimental, influences on the mental well-being of Black South Africans residing in Agincourt.

We did not find evidence that remaining in a dissolved marital status, remaining unmarried, or entering a new marriage impacts depressive symptoms for women. Regarding marital dissolution, past studies have also found that the negative impacts are stronger in the short term than the long term (De Leon et al., 2009; Harlow et al., 1991). For aging adult women in Agincourt, the experience of divorce and especially widowhood is common. Women may be particularly mentally prepared for the experience of widowhood without the expectation of remarriage, and this preparedness may benefit their depressive outcomes in the long run (but perhaps not as much in the short run). Moreover, women in Agincourt often reap support from relatives (Jennings et al., 2020). The normative nature of these experiences combined with strong kin ties may help women to navigate the stressors of marital dissolution, and the effects of this process may be more observable for women who remained in dissolved statuses between waves than for those who recently entered those statuses. Likewise, women in this setting often take on financial responsibilities for themselves and their families (Hosegood et al., 2009), which may make them more resilient to being in a long-term, unmarried status. The social support outside of marriage and the financial role that women hold may also help to explain why a stable marriage was not significantly more (or less) advantageous than having entered a new marriage among women in our sample.

Unlike women, men who entered a new marriage, remained in a dissolved marital status, or remained never married between waves were found to experience greater depressive symptoms than their stably married counterparts. This complements work from both Malawi and Western settings that has suggested that men may benefit more from marriage (and suffer more from non-married statuses) than women (Hsu & Barrett, 2020; Marks, 1996; Myroniuk et al., 2021). Although we expected that men in Agincourt would not experience as much benefit from marriage as in settings where they may be better able to fulfill the financial responsibilities of marriage that are tied to their identity, our results suggest that men in Agincourt do reap mental health benefits from stable marriage. Interestingly, we found modest evidence that men who entered a new marriage between waves experienced greater depressive symptoms than those who remained married between waves. It may be that the quality of support that men receive from a long-time spouse is greater than what they receive from a more recent spouse. Further research is needed to confirm this association and to decipher the mechanisms at play.

Although previous research has suggested that a loss of economic resources can explain much of the toll that marital dissolution takes on women in Western settings (Smock et al., 1999; Strohschein et al., 2005), the null results from our analyses are not surprising given women’s economic independence in this setting. Our null results may be due to the separation of economic well-being from marriage for women in Agincourt. The results for men are perhaps more surprising, given the importance of economic status for their identity (Barker & Ricardo, 2005; Bigombe & Khadiagala, 2003; Fry et al., 2019; King & Stone, 2010). It is also possible that the null results are due to the population in Agincourt being very poor, and so movement across wealth quintiles may not be meaningful for the ways in which marital status can impact depressive outcomes.

Our investigation faces limitations that should be considered in interpreting these results. First, we are unable to assess full marital histories with the HAALSI data. While we do adjust for past marital experiences using a control variable to indicate that respondents’ current marriage is not their first, being able to account for potentially complex marital histories would be ideal (Hosegood et al., 2009; Reniers, 2003). For example, the depressive symptoms experienced in each marital status may be dependent on the length of time spent in that status (Barrett, 2000; Sasson & Umberson, 2013; Schaan, 2013), but we lack marital history data to determine these effects. A second, related, limitation is that the data we use span only about four years, limiting our ability to assess whether the detrimental impact of marital dissolution on depressive symptoms dissipates in the long term (Barrett, 2000; Schaan, 2013; Strohschein et al., 2005). Third, there may be differences in the ways in which men and women respond to the CES-D survey items (Adams et al., 2020), which could have impacted the differences we find between women and men. Fourth, our analyses are limited to those who did not attrite between waves of data collection. Twelve percent of the HAALSI cohort who was interviewed in wave 1 had died by wave 2, and some deaths may be related to depression (Ben-Arie et al., 2018; Haas et al., 2020; Pim Cuijpers et al., 2014; Walker et al., 2015). However, we do not currently have information on cause of death among this sample; analyses that account for this in the future could be fruitful. We do include supplemental tables that display results from our analyses when weighted for mortality and attrition, which show very little difference from the results we presented here (see Supplemental Tables 4–6).

Overall, our findings provide important new insight into the interrelationships between marital transitions, economic and social resources, and mental health in a SSA setting. Our study uniquely evaluates these relationships in the context of racialized apartheid era policies, which resulted in the fragmentation of the Black African family. In our sample, consisting entirely of Black South Africans in middle and late adulthood, we find compelling evidence that the mental health benefits of a stable marriage are conferred to this population, and especially to men. At the same time, our study offers insight into consequences of the documented impact of colonial and apartheid policies and their legacy on increased marital dissolution (Budlender & Lund, 2011; Hosegood et al., 2009), highlighting that marital dissolution among Black South Africans may result in diminished mental well-being. Public health policies and programs geared toward mental health promotion should consider the impact of marital transitions, and especially marital dissolution, among middle-aged and older adults. Future population-level investigations focused on aging communities should also consider additional context-specific intermediary influences that buffer depressive symptoms as a function of marital dissolution.

## Supplementary Material

Supplemental Tables

## Figures and Tables

**Table 1:** Descriptive statistics (Means/proportions and standard deviations) of the analytic sample.

	Women (n = 2,315)	Men (n = 1,861)	Range	Gender Difference
Depressive symptoms wave 2	0.06 (1.00)	−0.07 (1.00)	−3.11– 3.85	0.13 ***
***Marital experiences***				
Remained married	0.31	0.60	0–1	−0.29 ***
Entered new marriage	0.02	0.04	0–1	−0.02 ***
Remained separated/divorced	0.09	0.09	0–1	0.00
Remained widowed	0.42	0.10	0–1	0.32 ***
Marital dissolution	0.14	0.12	0–1	0.02 ***
Remained never married	0.02	0.05	0–1	−0.03 ***
***Economic Resources***				
Decline in wealth between waves	0.05 (0.80)	0.07 (0.78)	−1 – 1	−0.02 **
***Controls (baseline)***				
Depressive symptoms wave 1	1.45 (1.65)	1.33 (1.47)	0–8	0.12 ***
Age	61.33 (12.66)	61.65 (12.44)	40–112	−0.32 *
Married more than once	0.08	0.38	0–1	−0.30 ***
Number of children	4.33 (2.19)	4.46 (2.52)	0–8	−0.13 ***
Comorbidity	0.71	0.62	0–1	0.09 ***
HIV positive	0.24	0.23	0–1	0.01
Education				
No formal	0.49	0.39	0–1	0.10 ***
Primary	0.33	0.36	0–1	−0.03 ***
Secondary	0.18	0.25	0–1	−0.07 ***
Employment				
Employed	0.13	0.17	0–1	−0.04 ***
Homemaker/home ‘manager’	0.13	0.08	0–1	0.05 ***
Retired	0.18	0.23	0–1	−0.05 ***
Not working	0.56	0.52	0–1	0.04 ***
Wealth index, quintiles	2.99 (1.41)	3.01 (1.43)	1–5	−0.02
Household size (top coded 8)	5.00 (2.27)	4.74 (2.43)	1–8	0.26 ***
Born in South Africa	0.68	0.70 (0.46)	0–1	−0.02 ***

Two-tailed t-tests

+ p < .10

* p < .05

** P < .01

*** p < .001.

Source: Health and Aging in Africa: A Longitudinal Study of an INDEPTH Community in South Africa (HAALSI)

**Table 2:** OLS regression of Marital experiences predicting depressive symptoms.

	Model 1 Women		Model 2 Men	
Intercept	−0.51 **	(0.18)	−0.29	(0.20)
***Marital experiences***				
Remained married	REF		REF	
Entered new marriage	−0.05	(0.17)	0.23 +	(0.14)
Remained separated/divorced	0.11	(0.09)	0.31 **	(0.09)
Remained widowed	0.01	(0.06)	0.20 *	(0.10)
Marital dissolution	0.18 *	(0.07)	0.33 ***	(0.08)
Remained never married	−0.001	(0.16)	0.33 *	(0.14)
***Controls (baseline)***				
Depressive symptoms wave 1	0.04 **	(0.01)	0.01	(0.02)
Age	0.01 ***	(0.002)	0.004	(0.003)
Married more than once	−0.01	(0.08)	0.01	(0.05)
Number of children	−0.02	(0.01)	−0.01	(0.01)
Comorbidity	0.03	(0.05)	0.02	(0.05)
HIV positive	0.07	(0.06)	0.04	(0.07)
Education				
No formal	REF		REF	
Primary	−0.04	(0.05)	−0.12 +	(0.06)
Secondary	0.03	(0.08)	−0.27 ***	(0.07)
Employment				
Employed	0.01	(0.07)	−0.15 *	(0.07)
Homemaker/home ‘manager’	−0.10	(0.07)	−0.11	(0.09)
Retired	−0.16 **	(0.06)	−0.10	(0.06)
Not working	REF		REF	
Wealth index	−0.001	(0.02)	−0.01	(0.02)
Household size (top coded 8)	0.01	(0.01)	0.003	(0.01)
Born in South Africa	−0.05	(0.05)	0.08	(0.06)
*N*	*2,315*	*1,861*		

Beta coefficients and standard errors in parentheses; two-tailed t-tests

+ p < .10,

* p < .05

** P < .01

*** p < .001.

Source: Health and Aging in Africa: A Longitudinal Study of an INDEPTH Community in South Africa (HAALSI)

**Table 3:** OLS regression of Marital experiences predicting depressive symptoms, main and Moderating associations with decline in wealth.

	Model 1 Women		Model 2 Women		Model 3 Men		Model 4 Men	
Intercept	−0.51 **	(0.17)	−0.51 **	(0.18)	−0.29	(0.20)	−0.28	(0.20)
***Marital experiences and Interactions***								
Remained married	REF		REF		REF		REF	
Entered new marriage	−0.05	(0.17)	−0.06	(0.17)	0.23 +	(0.14)	0.24 +	(0.14)
Entered new marriage × decline in wealth			0.11	(0.22)			−0.03	(0.15)
Remained separated/divorced	0.11	(0.09)	0.11	(0.09)	0.32 ***	(0.09)	0.32 ***	(0.09)
Remained separated/divorced × decline in wealth			−0.08	(0.11)			0.14	(0.12)
Remained widowed	0.01	(0.06)	0.01	(0.06)	0.21 *	(0.10)	0.20 *	(0.10)
Remained widowed × decline in wealth			−0.02	(0.07)			−0.04	(0.10)
Marital dissolution	0.18 *	(0.07)	0.18 *	(0.07)	0.34 ***	(0.08)	0.34 ***	(0.08)
Marital dissolution × decline in wealth			0.13	(0.09)			0.0002	(0.09)
Never married	0.0004	(0.16)	0.01	(0.16)	0.33 *	(0.14)	0.33 *	(0.14)
Never married × decline in wealth			−0.11	(0.18)			0.07	(0.14)
Decline in wealth	−0.02	(0.03)	−0.02	(0.05)	0.01	(0.03)	0.0002	(0.04)
***Controls (baseline)***								
Depressive symptoms wave 1	0.04 **	(0.01)	0.04 **	(0.01)	0.01	(0.02)	0.01	(0.02)
Age	0.01 ***	(0.002)	0.01 ***	(0.002)	0.003	(0.003)	0.003	(0.003)
Married more than once	0.01	(0.08)	−0.01	(0.08)	0.01	(0.05)	0.01	(0.05)
Number of children	−0.02	(0.01)	−0.02	(0.01)	−0.01	(0.01)	−0.01	(0.01)
Comorbidity	0.03	(0.05)	0.03	(0.05)	0.02	(0.05)	0.02	(0.05)
HIV positive	0.07	(0.06)	0.06	(0.06)	0.04	(0.07)	0.04	(0.07)
Education								
No formal	REF		REF		REF		REF	
Primary	−0.04	(0.05)	−0.04	(0.05)	−0.12 *	(0.06)	−0.12 +	(0.06)
Secondary	0.03	(0.07)	0.03	(0.07)	−0.28 ***	(0.07)	−0.28 ***	(0.07)
Employment								
Employed	0.004	(0.07)	0.003	(0.07)	−0.15 *	(0.07)	−0.15 *	(0.07)
Homemaker/home ‘manager’	−0.09	(0.07)	−0.10	(0.07)	−0.11	(0.09)	−0.12	(0.09)
Retired	−0.16 **	(0.06)	−0.16 **	(0.06)	−0.10	(0.06)	−0.10	(0.06)
Not working	REF		REF		REF		REF	
Household size (top coded 8)	0.01	(0.01)	0.01	(0.01)	0.002	(0.01)	0.002	(0.01)
Born in South Africa	−0.05	(0.05)	−0.05	(0.05)	0.07	(0.06)	0.07	(0.06)
*N*	*2,315*		*2,315*		*1,861*		*1,861*	

Beta coefficients and standard errors in parentheses; two-tailed t-tests

+ p < .10

* p < .05

** P < .01

*** p < .001.

Source: Health and Aging in Africa: A Longitudinal Study of an INDEPTH Community in South Africa (HAALSI)

## Footnotes

1 Measures indicating decline in wealth between waves and whether respondent was born in South Africa are not significantly different for men and women in a sample based on listwise deletion of missing cases (see Supplemental Table 1).

2 Entering a new marriage is not significantly associated with depressive symptoms for men in models estimated either using listwise deletion or using imputation with weights for mortality and other attrition (see Supplemental Tables 2 and 5).
