# Supplementary material for: Marital experiences and depressive symptoms among older adults in rural South Africa
Source: SSM Ment Health. 2022 Dec;2:100083. doi: 10.1016/j.ssmmh.2022.100083 (PMC9581082; doi:10.1016/j.ssmmh.2022.100083)
Supplement: Multimedia component 1 [file mmc1.docx]

**Supplemental Tables**

Supplemental Table 1. Descriptive Statistics (Means/Proportions and Standard Deviations) of the Analytic Sample, No Imputation of Missing values

|  | **Women**  **(n=1,915)** | **Men**  **(n=1,421)** | **Range** | **Gender Difference** |
| --- | --- | --- | --- | --- |
| Depressive symptoms wave 2 | 0.05 (1.01) | -0.10 (0.99) | -1.55 – 3.86 | 0.15 *** |
| ***Marital experiences*** |  |  |  |  |
| Remained married | 0.32 | 0.66 | 0 – 1 | -0.34 *** |
| Entered new marriage | 0.01 | 0.03 | 0 – 1 | -0.02 ** |
| Remained separated/divorced | 0.09 | 0.07 | 0 – 1 | 0.02 |
| Remained widowed | 0.42 | 0.10 | 0 – 1 | 0.32 *** |
| Marital dissolution | 0.14 | 0.11 | 0 – 1 | 0.03 * |
| Remained never married | 0.02 | 0.03 | 0 – 1 | -0.01 ** |
| ***Moderator*** |  |  |  |  |
| Change in wealth between waves | 0.05 (0.80) | 0.07 (0.78) | -1 – 1 | -0.02 |
| ***Controls (baseline)*** |  |  |  |  |
| Depressive symptoms wave 1 | 1.40 (1.62) | 1.27 (1.43) | 0 – 8 | 0.13 * |
| Age | 60.82 (12.16) | 62.01 (12.12) | 40 – 112 | -1.19 ** |
| Married more than once at baseline | 0.08 | 0.38 | 0 – 1 | -0.30 *** |
| Number of children | 4.40 (2.17) | 4.62 (2.47) | 0 – 8 | -0.22 ** |
| Comorbidity | 0.71 | 0.62 | 0 – 1 | 0.09 *** |
| HIV positive | 0.24 | 0.23 | 0 – 1 | 0.01 |
| Education |  |  |  |  |
| No formal | 0.48 | 0.38 | 0 – 1 | 0.10 *** |
| Primary | 0.35 | 0.38 | 0 – 1 | -0.03 + |
| Secondary | 0.18 | 0.24 | 0 – 1 | -0.06 *** |
| Employment |  |  |  |  |
| Employed | 0.14 | 0.18 | 0 – 1 | -0.04 *** |
| Homemaker/home ‘manager’ | 0.13 | 0.09 | 0 – 1 | 0.04 *** |
| Retired | 0.19 | 0.23 | 0 – 1 | -0.04 ** |
| Not working | 0.54 | 0.50 | 0 – 1 | 0.04 * |
| Wealth index, quintiles | 3.02 (1.40) | 3.05 (1.43) | 1 – 5 | -0.02 |
| Household size (top coded 8) | 5.04 (2.26) | 4.84 (2.44) | 1 – 8 | 0.20 * |
| Born in South Africa | 0.69 | 0.70 | 0 – 1 | -0.01 |

two-tailed t-tests, + p<.10 , * p<.05, **P<.01, *** p<.001

Source: Health and Aging in Africa: A Longitudinal Study of an INDEPTH Community in South Africa (HAALSI)

Supplemental Table 2. OLS Regression of Marital Experiences Predicting Depressive Symptoms, No Imputation of Missing values

|  | Model 1  Women | |  | Model 2  Men | |
| --- | --- | --- | --- | --- | --- |
| Intercept | -0.66 *** | (0.19) | -0.37 + | | (0.21) |
| ***Marital experiences*** |  |  |  | |  |
| Remained married | REF | | REF | | |
| Entered new marriage | -0.16 | (0.19) | 0.14 | | (0.16) |
| Remained separated/divorced | 0.14 | (0.09) | 0.30 ** | | (0.11) |
| Remained widowed | 0.002 | (0.06) | 0.19 + | | (0.10) |
| Marital dissolution | 0.19 * | (0.08) | 0.29 *** | | (0.09) |
| Remained never married | -0.09 | (0.17) | 0.37 * | | (0.16) |
| ***Controls (baseline)*** |  |  |  | |  |
| Depressive symptoms wave 1 | 0.05 ** | (0.01) | 0.01 | | (0.02) |
| Age | 0.01 *** | (0.003) | 0.01 + | | (0.003) |
| Married more than once | -0.02 | (0.08) | 0.04 | | (0.06) |
| Number of children | -0.02 | (0.01) | -0.01 | | (0.01) |
| Comorbidity | 0.02 | (0.05) | 0.01 | | (0.06) |
| HIV positive | 0.03 | (0.06) | 0.07 | | (0.07) |
| Education |  |  |  | |  |
| No formal | REF | | REF | | |
| Primary | -0.02 | (0.06) | -0.10 | | (0.07) |
| Secondary | 0.09 | (0.08) | -0.28 *** | | (0.08) |
| Employment |  |  |  | |  |
| Employed | -0.003 | (0.07) | -0.13 + | | (0.07) |
| Homemaker/home ‘manager’ | -0.14 * | (0.07) | -0.18 + | | (0.10) |
| Retired | -0.20 ** | (0.07) | -0.11 | | (0.07) |
| Not working | REF | | REF | | |
| Wealth index | -0.01 | (0.02) | -0.01 | | (0.02) |
| Household size (top coded 8) | 0.02 | (0.01) | 0.01 | | (0.01) |
| Born in South Africa | -0.07 | (0.06) | 0.06 | | (0.07) |
| *N* | *1,915* | | *1,421* | | |

Beta coefficients and standard errors in parentheses; two-tailed t-tests, + p<.10 , * p<.05, **P<.01, *** p<.001

Source: Health and Aging in Africa: A Longitudinal Study of an INDEPTH Community in South Africa (HAALSI)

Supplemental Table 3. OLS Regression of Marital Experiences Predicting Depressive Symptoms, Main and Moderating Associations with Decline in Wealth, No imputation of missing values

|  | Model 1  Women | | Model 2  Women | | Model 3  Men | | Model 4  Men | |
| --- | --- | --- | --- | --- | --- | --- | --- | --- |
| Intercept | -0.68 *** | (0.19) | -0.68 *** | (0.19) | -0.38 + | (0.21) | -0.39 + | (0.21) |
| ***Marital experiences*** |  |  |  |  |  |  |  |  |
| Remained married | REF | | REF | | REF | | REF | |
| Entered new marriage | -0.15 | (0.19) | -0.18 | (0.20) | 0.14 | (0.16) | 0.14 | (0.16) |
| X Change in wealth |  |  | 0.27 | (0.25) |  |  | 0.06 | (0.19) |
| Remained separated/divorced | 0.14 | (0.09) | 0.14 | (0.09) | 0.30 ** | (0.11) | 0.31 ** | (0.11) |
| X Change in wealth |  |  | -0.11 | (0.11) |  |  | 0.16 | (0.13) |
| Remained widowed | 0.004 | (0.06) | 0.01 | (0.06) | 0.19 + | (0.10) | 0.19 + | (0.10) |
| X Change in wealth |  |  | 0.001 | (0.07) |  |  | -0.08 | (0.11) |
| Marital dissolution | 0.19 * | (0.08) | 0.18 * | (0.08) | 0.30 *** | (0.09) | 0.29 *** | (0.09) |
| X Change in wealth |  |  | 0.14 | (0.09) |  |  | 0.01 | (0.11) |
| Remained never married | -0.09 | (0.17) | -0.04 | (0.18) | 0.37 * | (0.16) | 0.39 * | (0.16) |
| X Change in wealth |  |  | -0.23 | (0.21) |  |  | 0.13 | (0.17) |
| Change in wealth |  |  | -0.04 | (0.05) | 0.002 | (0.03) | -0.01 | (0.04) |
| ***Controls (baseline)*** |  |  |  |  |  |  |  |  |
| Depressive symptoms wave 1 | 0.05 ** | (0.01) | 0.05 ** | (0.01) | 0.01 | (0.02) | 0.01 | (0.02) |
| Age | 0.01 *** | (0.003) | 0.01 *** | (0.003) | 0.01 + | (0.003) | 0.01 + | (0.003) |
| Married more than once | -0.02 | (0.08) | -0.02 | (0.08) | 0.04 | (0.06) | 0.04 | (0.06) |
| Number of children | -0.02 | (0.01) | -0.02 | (0.01) | -0.01 | (0.01) | -0.01 | (0.01) |
| Comorbidity | 0.02 | (0.05) | 0.02 | (0.05) | 0.003 | (0.06) | 0.01 | (0.06) |
| HIV positive | 0.04 | (0.06) | 0.03 | (0.06) | 0.07 | (0.07) | 0.07 | (0.07) |
| Education |  |  |  |  |  |  |  |  |
| No formal | REF | | REF | | REF | | REF | |
| Primary | -0.03 | (0.06) | -0.03 | (0.06) | -0.11 | (0.07) | -0.10 | (0.07) |
| Secondary | 0.09 | (0.08) | 0.08 | (0.08) | -0.29 *** | (0.08) | -0.29 *** | (0.08) |
| Employment |  |  |  |  |  |  |  |  |
| Employed | -0.01 | (0.07) | -0.01 | (0.07) | -0.13 + | (0.07) | -0.13 + | (0.08) |
| Homemaker/home ‘manager’ | -0.14 * | (0.07) | -0.15 * | (0.07) | -0.18 + | (0.10) | -0.18 + | (0.10) |
| Retired | -0.20 ** | (0.06) | -0.20 ** | (0.06) | -0.11 | (0.07) | -0.11 | (0.07) |
| Not working | REF | | REF | | REF | | REF | |
| Household size (top coded 8) | 0.02 | (0.01) | 0.02 | (0.01) | 0.01 | (0.01) | 0.01 | (0.01) |
| Born in South Africa | -0.07 | (0.06) | -0.07 | (0.06) | 0.05 | (0.06) | 0.05 | (0.06) |
| *N* | *1,915* | | *1,915* | | *1,421* | | *1,421* | |

Beta coefficients and standard errors in parentheses; two-tailed t-tests , + p<.10 , * p<.05, **P<.01, *** p<.001

Source: Health and Aging in Africa: A Longitudinal Study of an INDEPTH Community in South Africa (HAALSI)

Supplemental Table 4. Descriptive Statistics (Means/Proportions and Standard Deviations) of the Analytic Sample, Weighted for Mortality and Attrition

|  | **Women**  **(n=2,315)** | **Men**  **(n=1,861)** | **Range** | **Gender Difference** |
| --- | --- | --- | --- | --- |
| Depressive symptoms wave 2 | 0.06 (1.08) | -0.05 (1.12) | -3.11 – 3.85 | 0.11 *** |
| ***Marital experiences*** |  |  |  |  |
| Remained married | 0.29 | 0.59 | 0 – 1 | -0.30 *** |
| Entered new marriage | 0.02 | 0.04 | 0 – 1 | -0.02 *** |
| Remained separated/divorced | 0.09 | 0.09 | 0 – 1 | 0.00 |
| Remained widowed | 0.44 | 0.11 | 0 – 1 | 0.33 *** |
| Marital dissolution | 0.14 | 0.12 | 0 – 1 | 0.02 *** |
| Remained never married | 0.02 | 0.05 | 0 – 1 | -0.03 *** |
| ***Moderator*** |  |  |  |  |
| Change in wealth between waves | 0.05 (0.87) | 0.07 (0.87) | -1 – 1 | -0.02 * |
| ***Controls (baseline)*** |  |  |  |  |
| Depressive symptoms wave 1 | 1.50 (1.83) | 1.39 (1.70) | 0 – 8 | 0.11 *** |
| Age | 62.13 (14.14) | 62.64 (14.49) | 40 – 112 | -0.51 *** |
| Married more than once at baseline | 0.08 | 0.38 | 0 – 1 | -0.30 *** |
| Number of children | 4.29 (2.38) | 4.43 (2.85) | 0 – 8 | -0.14 *** |
| Comorbidity | 0.72 | 0.63 | 0 – 1 | 0.09 *** |
| HIV positive | 0.23 | 0.23 | 0 – 1 | 0.00 |
| Education |  |  |  |  |
| No formal | 0.50 | 0.41 | 0 – 1 | 0.09 *** |
| Primary | 0.33 | 0.36 | 0 – 1 | -0.03 *** |
| Secondary | 0.17 | 0.23 | 0 – 1 | -0.06 *** |
| Employment |  |  |  |  |
| Employed | 0.13 | 0.17 | 0 – 1 | -0.04 *** |
| Homemaker/home ‘manager’ | 0.12 | 0.08 | 0 – 1 | 0.04 *** |
| Retired | 0.19 | 0.23 | 0 – 1 | -0.04 *** |
| Not working | 0.56 | 0.52 | 0 – 1 | 0.04 *** |
| Wealth index, quintiles | 2.99 (1.53) | 2.98 (1.61) | 1 – 5 | 0.01 |
| Household size (top coded 8) | 4.98 (2.46) | 4.69 (2.75) | 1 – 8 | 0.29 *** |
| Born in South Africa | 0.69 | 0.71 | 0 – 1 | -0.02 *** |

two-tailed t-tests, + p<.10 , * p<.05, **P<.01, *** p<.001

Source: Health and Aging in Africa: A Longitudinal Study of an INDEPTH Community in South Africa (HAALSI)

Supplemental Table 5. OLS Regression of Marital Experiences Predicting Depressive Symptoms, Weighted for Mortality and Attrition

|  | Model 1  Women | |  | Model 2  Men | |
| --- | --- | --- | --- | --- | --- |
| Intercept | -0.50 ** | (0.18) | -0.26 | | (0.20) |
| ***Marital experiences*** |  |  |  | |  |
| Remained married | REF | | REF | | |
| Entered new marriage | -0.03 | (0.16) | 0.21 | | (0.14) |
| Remained separated/divorced | 0.11 | (0.09) | 0.30 ** | | (0.09) |
| Remained widowed | 0.02 | (0.06) | 0.20 * | | (0.10) |
| Marital dissolution | 0.18 * | (0.07) | 0.34 *** | | (0.08) |
| Remained never married | -0.01 | (0.16) | 0.29 * | | (0.14) |
| ***Controls (baseline)*** |  |  |  | |  |
| Depressive symptoms wave 1 | 0.03 ** | (0.01) | 0.01 | | (0.02) |
| Age | 0.01 *** | (0.003) | 0.004 | | (0.003) |
| Married more than once | -0.01 | (0.08) | -0.002 | | (0.05) |
| Number of children | -0.02 | (0.01) | -0.01 | | (0.01) |
| Comorbidity | 0.03 | (0.05) | 0.02 | | (0.05) |
| HIV positive | 0.07 | (0.06) | 0.02 | | (0.07) |
| Education |  |  |  | |  |
| No formal | REF | | REF | | |
| Primary | -0.04 | (0.05) | -0.13 * | | (0.06) |
| Secondary | 0.03 | (0.08) | -0.29 *** | | (0.08) |
| Employment |  |  |  | |  |
| Employed | 0.003 | (0.07) | -0.15 * | | (0.07) |
| Homemaker/home ‘manager’ | -0.10 | (0.07) | -0.11 | | (0.09) |
| Retired | -0.16 * | (0.06) | -0.09 | | (0.06) |
| Not working | REF | | REF | | |
| Wealth index | -0.002 | (0.02) | -0.01 | | (0.02) |
| Household size (top coded 8) | 0.01 | (0.01) | 0.004 | | (0.01) |
| Born in South Africa | -0.04 | (0.05) | 0.09 | | (0.06) |
| *N* | *2,315* | | *1,861* | | |

Beta coefficients and standard errors in parentheses; two-tailed t-tests, + p<.10 , * p<.05, **P<.01, *** p<.001

Source: Health and Aging in Africa: A Longitudinal Study of an INDEPTH Community in South Africa (HAALSI)

Supplemental Table 6. OLS Regression of Marital Experiences Predicting Depressive Symptoms, Main and Moderating Associations with Decline in Wealth, Weighted for Mortality and Attrition

|  | Model 1  Women | | Model 2  Women | | Model 3  Men | | Model 4  Men | |
| --- | --- | --- | --- | --- | --- | --- | --- | --- |
| Intercept | -0.51 ** | (0.18) | -0.51 ** | (0.18) | -0.27 | (0.20) | -0.25 | (0.21) |
| ***Marital experiences*** |  |  |  |  |  |  |  |  |
| Remained married | REF | | REF | | REF | | REF | |
| Entered new marriage | -0.03 | (0.16) | -0.05 | (0.16) | 0.21 | (0.14) | 0.21 | (0.14) |
| X Change in wealth |  |  | 0.11 | (0.22) |  |  | -0.06 | (0.15) |
| Remained separated/divorced | 0.11 | (0.09) | 0.11 | (0.09) | 0.30 ** | (0.09) | 0.30 ** | (0.09) |
| X Change in wealth |  |  | -0.08 | (0.11) |  |  | 0.15 | (0.12) |
| Remained widowed | 0.02 | (0.06) | 0.02 | (0.06) | 0.20 * | (0.10) | 0.20 * | (0.10) |
| X Change in wealth |  |  | -0.02 | (0.06) |  |  | -0.05 | (0.10) |
| Marital dissolution | 0.18 * | (0.07) | 0.18 * | (0.07) | 0.34 *** | (0.08) | 0.34 *** | (0.08) |
| X Change in wealth |  |  | 0.12 | (0.09) |  |  | 0.03 | (0.09) |
| Remained never married | -0.01 | (0.16) | -0.002 | (0.16) | 0.29 * | (0.14) | 0.29 * | (0.14) |
| X Change in wealth |  |  | -0.08 | (0.18) |  |  | 0.05 | (0.15) |
| Change in wealth | -0.02 | (0.03) | -0.02 | (0.05) | 0.02 | (0.03) | 0.01 | (0.04) |
| ***Controls (baseline)*** |  |  |  |  |  |  |  |  |
| Depressive symptoms wave 1 | 0.03 ** | (0.01) | 0.04 ** | (0.01) | 0.01 | (0.02) | 0.01 | (0.02) |
| Age | 0.01 *** | (0.003) | 0.01 *** | (0.003) | 0.004 | (0.003) | 0.004 | (0.003) |
| Married more than once | -0.01 | (0.08) | -0.01 | (0.08) | -0.002 | (0.05) | -0.003 | (0.05) |
| Number of children | -0.02 | (0.01) | -0.02 | (0.01) | -0.01 | (0.01) | -0.01 | (0.01) |
| Comorbidity | 0.03 | (0.05) | 0.03 | (0.05) | 0.01 | (0.05) | 0.01 | (0.05) |
| HIV positive | 0.07 | (0.06) | 0.07 | (0.06) | 0.02 | (0.07) | 0.02 | (0.07) |
| Education |  |  |  |  |  |  |  |  |
| No formal | REF | | REF | | REF | | REF | |
| Primary | -0.04 | (0.05) | -0.04 | (0.05) | -0.14 * | (0.06) | -0.13 * | (0.06) |
| Secondary | 0.03 | (0.08) | 0.02 | (0.08) | -0.31 *** | (0.07) | -0.30 *** | (0.07) |
| Employment |  |  |  |  |  |  |  |  |
| Employed | 0.001 | (0.07) | 0.001 | (0.07) | -0.15 * | (0.07) | -0.15 * | (0.07) |
| Homemaker/home ‘manager’ | -0.09 | (0.07) | -0.10 | (0.07) | -0.12 | (0.09) | -0.12 | (0.09) |
| Retired | -0.16 * | (0.06) | -0.16 * | (0.06) | -0.09 | (0.06) | -0.09 | (0.06) |
| Not working | REF | | REF | | REF | | REF | |
| Household size (top coded 8) | 0.01 | (0.01) | 0.01 | (0.01) | 0.003 | (0.01) | 0.002 | (0.01) |
| Born in South Africa | -0.04 | (0.05) | -0.04 | (0.05) | 0.08 | (0.06) | 0.07 | (0.06) |
| *N* | *2,315* | | *2,315* | | *1,861* | | *1,861* | |

Beta coefficients and standard errors in parentheses; two-tailed t-tests , + p<.10 , * p<.05, **P<.01, *** p<.001

Source: Health and Aging in Africa: A Longitudinal Study of an INDEPTH Community in South Africa (HAALSI)
